# Supplementary material for: Social-Ecological Examination of Non-Consensual Sexting Perpetration among U.S. Adolescents
Source: Int J Environ Res Public Health. 2020 Dec 17;17(24):9477. doi: 10.3390/ijerph17249477 (PMC7766393; doi:10.3390/ijerph17249477)
Supplement: Supplementary file 1 [file ijerph-17-09477-s001.pdf]

Supplemental Table 1. Bivariate Correlations.

		**(1)**	**(2)**	**(3)**	**(4)**	**(5)**	**(6)**	**(7)**	**(8)**	**(9)**	**(10)**
**(1)**	Age (Years)	-									
**(2)**	Female	-0.03	-								
**(3)**	LGB	-0.02	0.19	-							
**(4)**	African American	0.04	0.01	-0.01	-						
**(5)**	White	0.01	-0.02	0.01	-0.71	-					
**(6)**	Other	-0.07	0.01	0.00	-0.44	-0.32	-				
**(7)**	Sexting Perp.	-0.01	-0.05	0.02	0.04	-0.04	-0.01	-			
**(8)**	Empathy	-0.01	-0.01	0.01	0.02	0.00	-0.02	-0.01	-		
**(9)**	Adult Support	0.03	-0.01	-0.03	-0.09	0.09	0.01	-0.03	-0.02	-	
**(10)**	Family Support	-0.01	0.01	-0.13	-0.12	0.14	-0.02	-0.07	0.00	0.45	-
**(11)**	Peer Support	-0.05	0.10	-0.02	-0.20	0.17	0.04	-0.03	-0.01	0.39	0.54
**(12)**	School Belonging	-0.01	-0.05	-0.10	-0.07	0.06	0.01	-0.02	0.01	0.47	0.28
**(13)**	Parental Monitoring	-0.07	0.05	-0.07	-0.05	0.05	0.01	-0.08	-0.02	0.28	0.42
**(14)**	Impulsivity	0.00	0.01	-0.02	-0.01	0.00	0.01	0.00	0.12	-0.02	0.02
**(15)**	Pornography	0.04	-0.01	0.02	-0.02	0.00	0.02	0.05	0.00	0.01	0.03
**(16)**	No. Dating Partners	-0.02	-0.17	0.04	0.24	-0.22	-0.03	0.08	0.00	-0.05	-0.14
**(17)**	Sexual Activity	0.00	0.01	0.01	0.00	0.01	-0.01	-0.02	0.00	0.03	0.05
**(18)**	Risky Sexual Act.	0.00	0.01	-0.02	0.01	-0.04	0.04	0.00	-0.10	0.05	0.00
**(19)**	Alcohol and Drugs	0.11	-0.03	0.14	0.02	0.03	-0.06	0.13	0.00	-0.14	-0.15
**(20)**	Homophobic Perp.	0.03	-0.19	0.00	0.10	-0.06	-0.05	0.18	0.02	-0.11	-0.09
**(21)**	Bullying Perp.	-0.02	-0.05	0.05	0.13	-0.10	-0.04	0.27	0.05	-0.11	-0.13
**(22)**	Delinquency	0.02	-0.05	0.10	0.02	-0.01	-0.01	0.19	0.01	-0.11	-0.15

(11)	(12)	(13)	(14)	(15)	(16)	(17)	(18)	(19)	(20)	(21)	(22)
-											
0.22	-										
0.22	0.22	-									
0.04	-0.01	0.02	-								
0.02	0.00	0.00	0.11	-							
-0.10	-0.05	-0.17	-0.02	0.01	-						
0.07	0.02	0.03	0.07	0.21	0.02	-					
0.01	0.00	-0.02	0.13	0.15	0.06	0.45	-				
-0.09	-0.17	-0.29	0.00	0.03	0.16	-0.04	0.01	-			
-0.07	-0.13	-0.18	0.02	0.00	0.15	0.00	0.01	0.23	-		
-0.07	-0.11	-0.23	0.04	0.00	0.11	-0.01	0.04	0.25	0.55	-	
-0.03	-0.17	-0.34	0.01	0.03	0.17	0.00	0.04	0.51	0.36	0.44	-
